# Quadruple Hydrogen Bond-Containing A-AB-A Triblock Copolymers: Probing the Influence of Hydrogen Bonding in the Central Block

**DOI:** 10.3390/molecules26154705

**Published:** 2021-08-03

**Authors:** Boer Liu, Xi Chen, Glenn A. Spiering, Robert B. Moore, Timothy E. Long

**Affiliations:** 1 Biodesign Center for Sustainable Macromolecular Materials and Manufacturing, School of Molecular Sciences, Arizona State University, Tempe, AZ 85281, USA; bliu117@asu.edu; 2 Department of Chemistry, Macromolecules Innovation Institute (MII), Virginia Tech, Blacksburg, VA 24061, USA; xichen4@vt.edu (X.C.); gaspiering@vt.edu (G.A.S.); rbmoore3@vt.edu (R.B.M.)

**Keywords:** quadruple hydrogen bonding, acrylic thermoplastic elastomer, supramolecular polymer, microphase separation

## Abstract

This work reveals the influence of pendant hydrogen bonding strength and distribution on self-assembly and the resulting thermomechanical properties of A-AB-A triblock copolymers. Reversible addition-fragmentation chain transfer polymerization afforded a library of A-AB-A acrylic triblock copolymers, wherein the A unit contained cytosine acrylate (CyA) or post-functionalized ureido cytosine acrylate (UCyA) and the B unit consisted of *n*-butyl acrylate (*n*BA). Differential scanning calorimetry revealed two glass transition temperatures, suggesting microphase-separation in the A-AB-A triblock copolymers. Thermomechanical and morphological analysis revealed the effects of hydrogen bonding distribution and strength on the self-assembly and microphase-separated morphology. Dynamic mechanical analysis showed multiple tan delta (δ) transitions that correlated to chain relaxation and hydrogen bonding dissociation, further confirming the microphase-separated structure. In addition, UCyA triblock copolymers possessed an extended modulus plateau versus temperature compared to the CyA analogs due to the stronger association of quadruple hydrogen bonding. CyA triblock copolymers exhibited a cylindrical microphase-separated morphology according to small-angle X-ray scattering. In contrast, UCyA triblock copolymers lacked long-range ordering due to hydrogen bonding induced phase mixing. The incorporation of UCyA into the soft central block resulted in improved tensile strength, extensibility, and toughness compared to the AB random copolymer and A-B-A triblock copolymer comparisons. This study provides insight into the structure-property relationships of A-AB-A supramolecular triblock copolymers that result from tunable association strengths.

## 1. Introduction

In contrast to chemically crosslinked thermoset rubbers, thermoplastic elastomers (TPEs) consist of thermoreversible physical crosslinks, which allow for recycling and reprocessing through melt-extrusion and injection-molding [1,2]. Poly(styrene-*b*-butadiene-*b*-styrene) (SBS) triblock copolymers as well as isoprene analogs and hydrogenated versions collectively serve as prominent TPEs in the automotive industry and common consumer products such as asphalt additives. This impact originates from similar mechanical properties to chemically crosslinked rubber counterparts as well as excellent thermal, oil, and abrasion resistance [3,4]. The glass transition temperatures (*T*_g_) of the poly(1,3-butadiene) and polystyrene blocks are −90 °C and 100 °C, respectively. Thus, the SBS TPE exhibits a wide working-temperature window between the two *T*_g_s. However, the triblock copolymer flows below 100 °C due to the partially miscible styrene and butadiene blocks, limiting the upper service temperature [4,5]. Alternatively, supramolecular strategies including H-bonding, electrostatic interactions, and pi-pi stacking offer routes to extend the upper service temperature due to the introduction of additional physical crosslinks into the hard domain [2,6,7]. Moreover, these specific noncovalent interactions, which impart enhanced non-covalent bond strengths compared to only van der Waals forces, improved the stiffness of polymers [8,9].

Nucleobase-containing polymers bearing specific hydrogen bonding recognition are a crucial design feature for tailoring supramolecular TPE properties [10]. In addition to the incorporation of thermo- and solvent-reversible crosslinks [11,12,13,14], the presence of nucleobase sites in TPEs afford novel properties such as self-healing and shape memory [15,16,17]. The physical properties of a nucleobase-functionalized polymer strongly depend on the association strength of nucleobases. Long et al. discovered that the blend of adenine-functionalized and thymine-functionalized A-B-A triblock copolymers exhibited an extended modulus plateau compared to the adenine or thymine triblock copolymer alone [18]. The improved thermomechanical properties resulted from the stronger association of the adenine-thymine pair compared to the significantly weaker self-associations. Structural modification and molecular design further allowed tunability of the association strength. Hailie et al. modified cytosine through the reaction of the primary amine with an isocyanate to afford the ureido cytosine (UCy) with quadruple hydrogen bonding (QHB) [19]. The UCy self-association constant was 2.5 × 10^5^ M^−1^, significantly higher than cytosine (Cy) (*ca.* 40 M^−1^) in chloroform (CHCl_3_) [19,20]. To further probe the effects of hydrogen bonding strength on the bulk morphology and mechanical properties, Long et al. reported UCy and Cy functionalized A-B-A triblock polymers and observed a 30 °C extended service window for the UCy-containing polymer compared to its Cy counterpart due to higher association strength of the UCy unit [21]. The increased upper service temperature afforded ureido cytosine acrylate (UCyA) copolymers as a novel high performance TPE.

Traditionally, A-B-A triblock copolymers represent the most common architecture for TPEs. Wang et al. designed A-B-A triblock copolymer using polybenzofulvene (PBF) as the hard A block and polyisoprene as the soft B block. The work extended the polymer service temperature to 145 °C owing to an exceptional high *T*_g_ of the PBF block, which is 40 °C higher than PS [7]. A-B-A triblock copolymer containing nucleobase on the pendant group demonstrated a significant reinforcement of thermomechanical properties and morphological orderings compared to the random copolymer analog [10,18]. With the development of advanced polymerization techniques, recent TPE research has investigated complex chain architectures, such as A-B-C triblock, A-BC-A triblock, pentablock, and star-shaped copolymers [6,13,22,23,24]. A-B-C triblock copolymers, which bear different external blocks, i.e., A and C, self-assembled into complex three-phase morphologies due to the presence of three different blocks. The morphology of ABC triblock copolymer is dependent on the chain length [22,25] and compatibility of A and C blocks [26]. Other researchers developed A-BC-A triblock copolymers, where they incorporated the hydrogen bonding containing-C unit into the central block to form weak crosslinks in the soft domain [27,28,29]. These crosslinks dissociated and associated dynamically during elongation, which effectively dissipated energy and prevented local stress concentration. As a result, the toughness increased over 100 times compared to the polymer in the absence of hydrogen bonding in the soft phase [27]. Yoshie et al. synthesized A-AB-A triblock copolymers using ring-opening metathesis polymerization (ROMP), where the A unit was ureidopyrimidone (UPy)-functionalized norbornene that formed self-complementary QHB, and B unit was the flexible poly(dodecanyl norbornene) [30]. The designed TPE exhibited tensile properties that were 36× toughness, 16× tensile strength, and 2.5× elongation of the A-B-A counterpart [30]. Their following work synthesized A-AB-A triblock copolymers with a varying number of A units (the hydrogen bonding unit) in the soft block. The authors observed a systematic increase of storage modulus with increasing A unit in the central block [31]. Despite the advantages of dynamic bonds in TPEs, the effects of physical crosslinking strength of the A unit on mechanical and morphological properties remain unexplored. Moreover, understanding the relationship between block interaction and physical crosslinking strength is crucial for predicting the 3D microstructure and tailoring the thermomechanical performances of TPEs.

Herein, we utilized Cy and UCy functional groups to design a library of A-AB-A triblock copolymers, where the A unit contained Cy or UCy and the B unit consisted of a flexible poly(*n*-butyl acrylate) (P*n*BA). This study detailed the synthesis and characterization of supramolecular triblock copolymers and explored the manipulation of hydrogen bonding strength and distribution in these copolymers for tunable physical properties. Reversible addition-fragmentation chain transfer (RAFT) copolymerization of CyA and *n*BA prepared a macro chain-transfer agent (macro-CTA) with a statistical distribution of monomers. Subsequently, chain extension using CyA afforded A-AB-A triblock copolymers containing two CyA end blocks. Finally, post-functionalization of the CyA triblock copolymer precursors yielded corresponding UCyA triblock copolymers with an identical distribution of the hydrogen bonding units, allowing for a direct comparison of solid-state properties. Systematic characterization of thermal, thermomechanical, and morphological properties revealed the synergy of mechanical properties and self-assembled microstructures. In addition, tensile testing assessed the effects of architecture on the mechanical properties of UCyA copolymers. The A-AB-A triblock copolymer exhibited improved overall tensile properties compared to the AB and A-B-A counterparts.

## 2. Results and Discussion

### 2.1. Synthesis of CyA and UCyA Triblock Copolymer

**Scheme 1:**
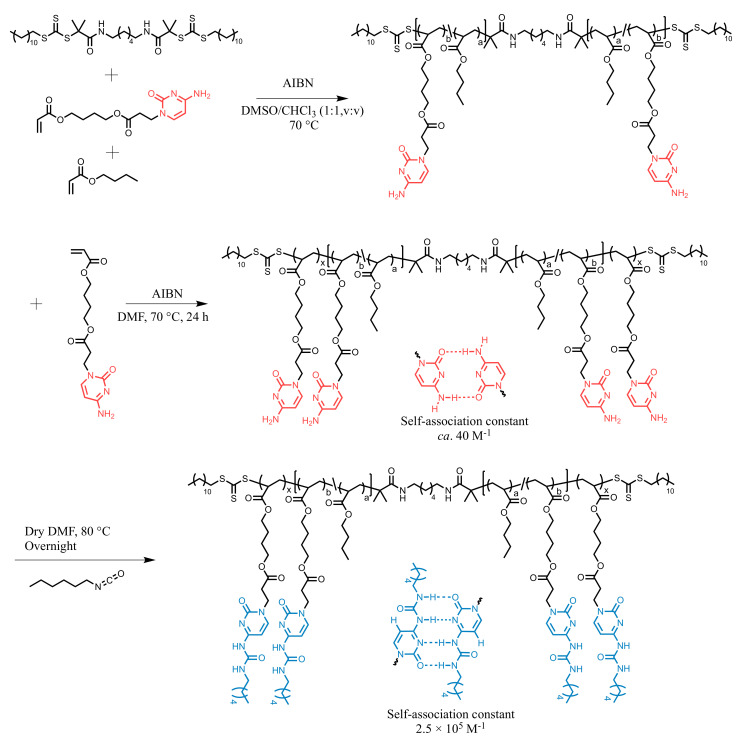
Synthesis of poly(CyA-*b*-(*n*BA-*co*-CyA)-*b*-CyA) and poly(UCyA-*b*-(*n*BA-*co*-UCyA)-*b*-UCyA) triblock copolymers using RAFT polymerization.

In the design of triblock copolymers, varying CyA/UCyA content in the central block and maintaining the chain length of each block enabled investigation of the effects of hydrogen bonding concentration and strength on mechanical and morphological properties. RAFT polymerization of *n*BA and CyA monomers yielded triblock copolymers with tunable chain lengths (Scheme 1 and Table 1). To generate polymers with a defined A-AB-A triblock architecture, a difunctional CTA, difunctional 2-(dodecylthiocarbonothioylthio)-2-methylpropionic acid (di-DDMAT) was selected due to its excellent compatibility with acrylic-based monomers [18,21]. A series of macro-CTAs, i.e., copolymers of *n*BA and CyA, were prepared. A solvent mixture of dimethyl sulfoxide (DMSO) and CHCl_3_ dissolved the reactants, and the resulting mixture maintained a homogeneous solution throughout the reaction. Dialyzing in a CH_3_OH/dichloromethane (CH_2_Cl_2_) mixture for two days and subsequently drying under a reduced pressure at room temperature effectively removed unreacted monomers and high-boiling solvents. Proton nuclear magnetic resonance (^1^H NMR) spectroscopy was the primary tool for determining molecular weight since the challenging solubility of the nucleobase functionalized copolymers limited utilization of size exclusion chromatography. The resonance at 7.4 ppm in Figure S1A corresponded to the chemical shift of a proton on the cytosine site and was utilized to normalize the proton integrations. The resonance at 0.8 ppm was associated with the methyl protons (3H) on *n*BA. The integral ratio between acrylic protons (3H) at 5.8–6.3 ppm and the sum of CyA and *n*BA protons yielded 97% conversion of the copolymerization. In addition, utilizing the integrals of CyA and *n*BA protons elucidated the fraction of CyA in the purified copolymer (4 mol %) (Figure S1B). Thus, monomer conversion of the copolymerization was calculated as described in the ESI. Finally, the degree of polymerization (DP) of each segment was calculated from the monomer feed and conversion (Table S1). The targeted DP was utilized for nomenclature and the general expression of poly(CyA_50_-*b*-(*n*BA_480_-*co*-CyA_20_)-*b*-CyA_50_) and poly(UCyA_50_-*b*-(*n*BA_480_-*co*-UCyA_20_)-*b*-UCyA_50_) was abbreviated as C_50_-*b*-(B_480_-*co-*C_20_)-*b*-C_50_ and U_50_-*b*-(B_480_-*co-*U_20_)-*b*-U_50_, respectively.

**Figure 1:**
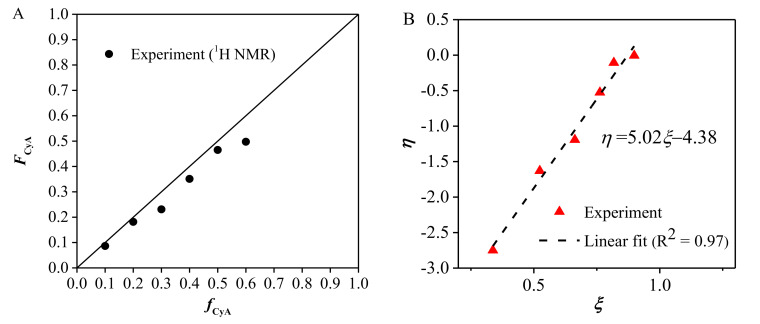
(**A**) Dependence of the instantaneous copolymer composition *F_CyA_* on the initial comonomer feed composition *f_CyA_*. The solid line represents a random copolymerization, where *r*_1_ = *r*_2_ = 1. (**B**) Calculation of reactivity ratios for the copolymerization of CyA and *n*BA using Kelen-Tüdös method.

Interestingly, the CyA monomer displayed a different rate of conversion than *n*BA during copolymerization, as evidenced from a significantly lower copolymer composition (*F_CyA_*) than the starting monomer feed ratio (*f_CyA_*) (Table S1), resulting in a compositional drift during copolymerization. In order to understand the origin of this phenomenon, reactivity ratios of *n*BA and CyA (*r_CyA_* and *r_nBA_*, respectively) were determined for the *n*BA and CyA copolymerization. The monomer conversion was analyzed at low conversion (<10%) to avoid concentration effects on reactivity ratios [32]. Since the copolymers exhibited a limited solubility in DMSO/CHCl_3_ solvent mixture when CyA content is higher than 60 mol %, the CyA monomer feed content was set to *f_CyA_* < 0.6. Figure 1A showed that *F_CyA_* at all investigated *f_CyA_* were located below the *F* = *f* line, i.e., an equal monomer reactivity ratio towards the propagating chain (*r*_1_ = *r*_2_ = 1). The lower CyA copolymer composition than the initial monomer feed ratio suggested a slower reactivity of CyA compared to *n*BA. To provide more in-depth insights on the origin of the compositional drift and relative reactivities of the two monomers, both Fineman-Ross (F-R) and Kelen-Tüdös (K-T) approaches were used to estimate *r_CyA_* and *r_nBA_*, according to the linear fitting of Equation (3) and (4), respectively. Both methods demonstrated excellent agreement in predicting the reactivity ratios of the CyA and *n*BA monomers, where the F-R method yielded *r_nBA_* = 1.03 and *r_CyA_* *=* 0.51 (Figure S4) and K-T yielded *r_nBA_* = 1.12 and *r_CyA_* = 0.64 (Figure 1A). *r_nBA_* (*r*_2_) ~ 1 indicated similar reactivity of *n*BA and CyA to the propagating *n*BA radical chain ends, while *r_CyA_* (*r*_1_) < 1 suggested that *n*BA has a preference towards adding to the CyA terminated propagating radical chain ends. The lower self-propagation compared to cross-propagation of CyA monomers was presumably attributed to its bulkier cytosine pendant unit, which inhibited the addition of another CyA monomer to a CyA-terminated radical [33]. Although the calculated reactivity ratios demonstrated that *n*BA propagates favorably at low conversions and thereby generating *n*BA-enriched polymer chains, it is of great interest to probe if the polymer displays a composition gradient along the polymer chain due to concentration effects at higher monomer conversions. Monitoring the *F_CyA_* with respect to conversion at equal initial monomer feed ratio (50:50) (Figure S5) revealed a relatively constant CyA composition from low (<10%) to high (>90%) conversion, which demonstrated a consistent polymer composition along the chain.

In the second step, the chain extension of the macro-CTA with CyA monomer yielded the CyA-containing triblock copolymer. An optimized ratio of CTA and initiator allowed the reaction to achieve >60% conversion in 24 h. ^1^H NMR spectroscopy of the reaction mixture yielded 65% monomer conversion, corresponding to an average of 49 CyA units for each external block, assuming an equal propagation rate on both chain ends. Dialysis of the reaction mixture in a CH_3_OH/CH_2_Cl_2_ mixture afforded a purified CyA-containing triblock copolymer. ^1^H NMR spectroscopic analysis of the purified product revealed 20 mol % of CyA incorporation in the triblock copolymer (Figure S2), which corresponds to an average of 50 CyA units on each external block (Table 1). This calculated value was closely aligned with the value calculated from monomer conversion.

Post-functionalization of CyA copolymer precursors using hexyl isocyanate afforded the corresponding UCyA triblock copolymers with an identical composition of hydrogen bonding units. An excess of isocyanate (1.5 eq) was introduced to the reaction to achieve quantitative conversion. A *N,N*-dimethylformamide (DMF)/DMSO mixture enabled the dissolution of the UCyA triblock copolymers to allow the reaction to proceed in a homogeneous fashion. ^1^H NMR spectroscopy indicated that the signal at 7.0–7.5 ppm, which originates from the amine hydrogen in the cytosine units, disappeared. In addition, two peaks located at 8.5–10.5 ppm corresponded to the urea protons, indicating the quantitative conversion of CyA- to UCyA-containing polymers (Figure S3). The resultant UCyA copolymers were light-yellow solids that were stiffer than their CyA analogs, presumably due to the stronger QHB for UCyA.

### 2.2. Thermal Analysis

**Figure 2:**
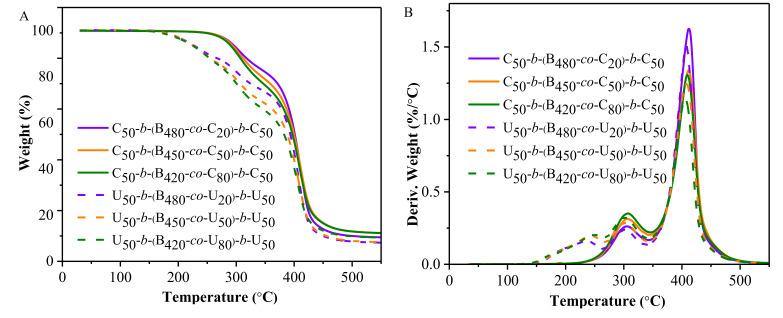
(**A**) TGA thermogram curves and (**B**) the corresponding weight derivative curves of CyA and UCyA triblock copolymers with varied amounts of hydrogen bonding unit.

Figure 2A reveals a variation in the thermal weight loss profiles of the triblock copolymers under N_2_ with increasing hydrogen bonding content. Derivatives of the weight loss profile in Figure 2B indicate a two-step weight loss mechanism in the CyA copolymers. The first step of the weight loss occurred at ~240 °C and was primarily attributed to the degradation of the butanediol diacrylate spacer. A second step occurred at ~300 °C, which involved the degradation of the acrylic backbone [13]. Thus, increasing concentrations of the hydrogen bonding sites in the central block resulted in a decreased *T*_d, 15 *wt.* %_, as presented in Table 1. For the UCyA copolymers, an additional weight loss step occurred at ~160 °C and involved the degradation of the urea bond at lower temperatures [34]. In addition, the *T*_d, 15 *wt.* %_ of CyA triblock copolymers were ~50 °C higher than the UCyA triblock copolymers (Table 1), demonstrating a potential higher thermal stability for CyA polymers under N_2_. However, it is worth noting that CyA copolymers crosslink at 130 °C as evidenced by isothermal rheological experiments under air atmosphere, which is possibly due to the reaction of amine with acrylate esters at elevated temperatures [34].

**Figure 3:**
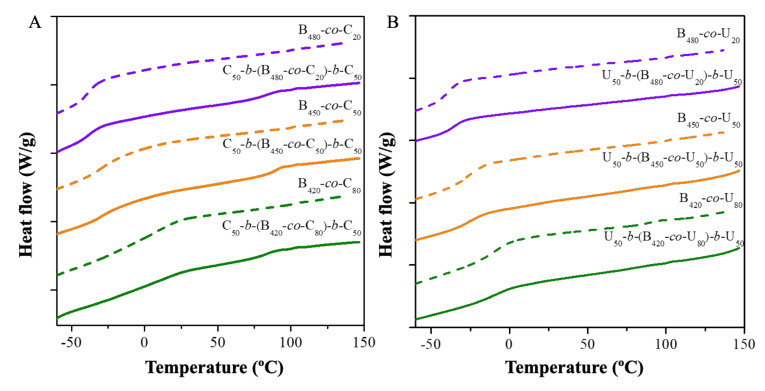
DSC thermograms of (**A**) CyA and (**B**) UCyA triblock copolymers with varied amounts of hydrogen bonding unit.

Differential scanning calorimetry (DSC) evaluated the glass transition temperatures of CyA and UCyA triblock copolymers and their corresponding random copolymer analogs with varied hydrogen bonding contents (Figure 3). The CyA random copolymers (dotted lines, Figure 3A) displayed a single transition step, with the *T*_g_ of the copolymer increasing with increasing CyA incorporation, which was consistent with our previous observations [34]. A single *T*_g_ confirmed the statistical sequence distribution of the random copolymers (Figure 3A). In addition, the glass transition step broadened as the CyA content increased, where hydrogen bonding formed between the randomly associated CyA units presumably resisted molecular mobility and expanded the timescale of molecular segmental motion within the polymer structure. In comparison, the CyA triblock copolymers displayed two well-resolved transitions within the testing range, suggesting a phase-separated morphology. The transition at lower temperatures agreed well with the corresponding random copolymer precursors and thus corresponded to the central random block. An additional higher temperature transition step at ~85 °C was associated with the external blocks. It is noteworthy that the *T*_g_ of the CyA external block was slightly lower compared with the *T*_g_ of a poly(CyA) homopolymer (~93 °C) [21], indicative of phase mixing that originated from interactions among the CyA units between the central and external blocks. Figure 3B depicts the thermal transition profiles of UCyA copolymers. Similar to CyA copolymers, the value of *T*_g_ agreed well with their random UCyA copolymer counterparts. However, DSC thermograms only revealed the central block transition within our testing regime. Lower thermal stability of the UCyA copolymers under N_2_ limited the upper testing temperature to 150 °C (degradation at ~160 °C). In fact, the slight increase of the heat flow near 140 °C was attributed to the onset of the thermal transition of the external blocks (Figure 3B) [21].

### 2.3. Thermomechanical Analysis

**Figure 4:**
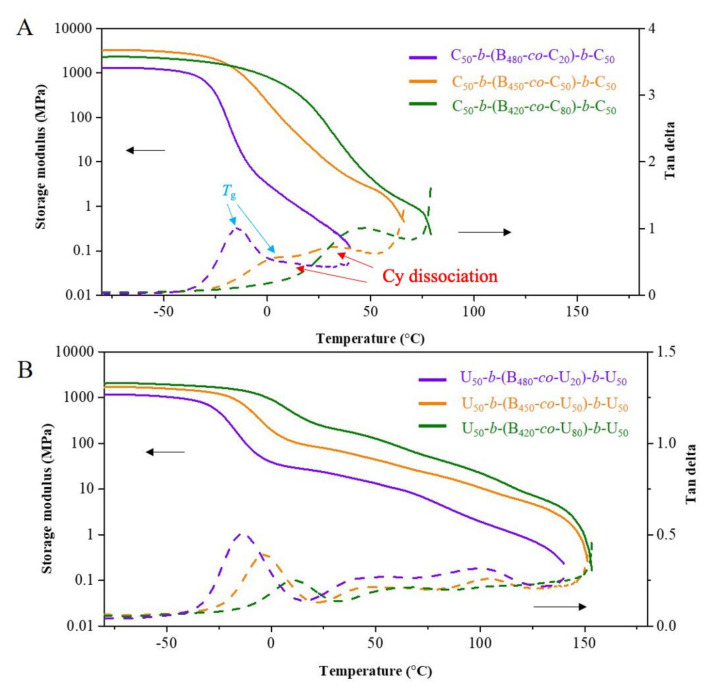
Dynamic mechanical temperature ramps of storage modulus and tan δ of the solution casted (**A**) CyA and (**B**) UCyA triblock copolymer films.

All CyA and UCyA copolymers afforded freestanding and optically transparent films through solvent-casting and annealing, indicating the absence of macrophase separation. Copolymers bearing a higher content of UCy showed poor solubility in polar solvents such as DMF or DMSO, but demonstrated good solubility in the mixture of DMF and DMSO. Therefore, the cosolvent system was used for solvent casting of all CyA and UCyA copolymers. Both DMSO and DMF are polar solvents that assist solvation of the polymers by dissociating strong hydrogen bonding interactions. In addition, utilizing high-boiling point solvents such as DMSO and DMF decreased solvent evaporation during annealing and thus facilitated polymer self-assembly [18]. Dynamic mechanical analysis (DMA) in Figure 4A,B evaluated the viscoelastic properties of the annealed CyA and UCyA copolymer films. A higher storage modulus was obtained with increased CyA/UCyA incorporation, indicating that hydrogen bonding improved the rigidity of the polymers. Additionally, both copolymer systems displayed multiple transitions verifying microphase-separated structures revealed using DSC. The first tan δ peak at lower temperature was related to the *T*_g_ of the soft central block. The value of *T*_g_ agreed well with the corresponding random copolymer precursors (Figure S6). In addition, the tan δ peak shifted to a higher temperature as CyA/UCyA content increased in the central block. The trend corroborated the observation in DSC (Table 1 and Figure 3), where increasing the central CyA/UCyA content in the central block restricted segmental motion of the polymer backbone due to the hydrogen bonding pendant group.

Further elevation of temperature resulted in the second tan δ peak for C_50_-*b*-(B_480_-*co-*C_20_)-*b*-C_50_ and C_50_-*b*-(B_450_-*co-*C_50_)-*b*-C_50_ due to the dissociation of Cy (Figure 4A). Increasing Cy incorporation elongated the terminal flow to higher temperatures, suggesting the formation of more robust networks due to the increased hydrogen bonding. Interestingly, C_50_-*b*-(B_420_-*co-*C_80_)-*b*-C_50_ displayed a single tan δ peak before terminal flow. Further investigation of its random precursor elucidated that the glass transition of the central block occurred at a similar temperature to Cy dissociation (Figure S10). In contrast, UCyA triblock copolymers exhibited multiple transitions on DMA (Figure 4B), which was different from CyA copolymers. Two tan δ transitions followed after the central block glass transition, which correspond to the dissociation of UCy in the soft and hard domains, respectively. The soft domains consist of UCyA units that were sparsely distributed in the P*n*BA matrix forming lightly packed crosslinks. On the contrary, the hard domains were composed of mostly UCyA that enabled the formation of densely packed crosslinks, thus dissociated at higher temperatures. Similarly, Long et al. observed an increasing terminal flow temperature when hydrogen bonding unit increased in the copolymer [21]. Additionally, all UCyA copolymers flowed at ~150 °C, suggesting minor effects of the central block on the terminal flow temperature. The onset glass transition temperature of the UCyA external block occurred at 140 °C, which was similar to the flow temperature observed in DMA results as observed in Figure 3B. The strong QHB in the UCyA enabled the retention of 3D networks until the temperature reached the external block *T*_g_, regardless of the hydrogen bonding concentration. Furthermore, UCyA copolymers increased the terminal flow temperature up to 100 °C compared to the CyA triblock copolymers. Such enhancement correlated to the highly oriented QHB for UCyA, which imparted higher association strength than the dimeric hydrogen bonding of Cy unit [34].

This section elucidates the influence of polymer architecture on mechanical properties using UCyA copolymer. The CyA A-AB-A triblock copolymers in this manuscript displayed a low modulus that presented challenges to prepare films using compression-molding, whereas the UCyA A-AB-A triblock copolymers formed mechanically robust films. Additionally, the UCyA triblock copolymer is more desirable as a TPE compared to the CyA triblock copolymer, as evidenced in Figure 4B. Specifically, the storage modulus of U_50_-*b*-(B_450_-*co-*U_50_)-*b*-U_50_ remained at the same magnitude from 10–30 °C, while DMA revealed a steep decrease in storage modulus for C_50_-*b*-(B_450_-*co-*C_50_)-*b*-C_50_ over this temperature range. Polymer samples with three different architectures: A-B-A triblock copolymer, A-AB-A, and AB copolymers were compared using tensile testing. These three samples have the same chain length (DP ~600) and the same UCy concentration (25 mol %) in each polymer chain. First, as shown in Figure S9, U_75_-*b*-B_450_-*b*-U_75_ suffered from cracking during film processing, where a concentrated UCy group in the outer block afforded highly oriented physical networks that result in a brittle mechanical property. As a result, U_75_-*b*-B_450_-*b*-U_75_ was too brittle to perform mechanical tests and tensile property was not obtained. In contrast, A-AB-A triblock copolymer containing additional dynamic crosslinks in the soft central block formed free-standing films that were suitable for tensile testing. Furthermore, the A-AB-A triblock copolymer demonstrated improved mechanical properties compared to the AB random copolymer counterpart, summarized in Table 2. The toughness of U_50_-*b*-(B_450_-*co-*U_50_)-*b*-U_50_ was twice of B_450_-*co-*U_150,_ with a simultaneous improvement of the elongation at break and ultimate stress that achieved 1.5 times and 1.3 times of the random polymer counterpart, respectively. The Young’s modulus of U_50_-*b*-(B_450_-*co-*U_50_)-*b*-U_50_ was lower than B_450_-*co-*U_150_. Comparably, DMA results confirmed the lower storage modulus of U_50_-*b*-(B_450_-*co-*U_50_)-*b*-U_50_ than B_450_-*co-*U_150_ (Figure S7) at room temperature, due to a lower concentration of UCy units in the central block U_50_-*b*-(B_450_-*co-*U_50_)-*b*-U_50_ that results in a lower *T*_g_ (~−3 °C) compared to that for B_450_-*co-*U_150_ (*T*_g_ ~47 °C) (Figure S7).

### 2.4. Morphological Characterization

**Figure 5:**
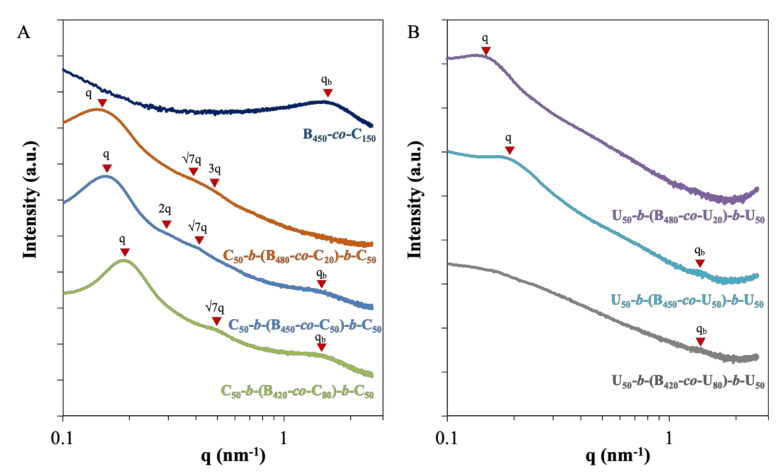
SAXS of solution casted (**A**) CyA and (**B**) UCyA triblock copolymer films with varied amounts of hydrogen bonding unit. Curves are shifted vertically for clarity.

Small angle X-ray scattering (SAXS) profiles in Figure 5 elucidated bulk morphologies and chain ordering of the CyA and UCyA triblock copolymers. CyA copolymers displayed a series of scattering maxima between *q* = 0.1 nm^−1^ and *q* = 1 nm^−1^, indicating a microphase-separated morphology that was driven by the ordering of hard and soft blocks. The periodic scattering maxima appeared at positions *q*, (2*q*), √7*q*, (3*q*), suggesting a hexagonally packed cylindrical morphology. The concentration of CyA in the central block showed an insignificant effect on the self-assembled morphology as all three CyA triblock copolymers displayed the hexagonally packed cylindrical morphology. The nearly identical chain length resulted in a similar volume fraction of the external blocks for all CyA copolymers, which provided an explanation for the compositional independent morphology [35,36]. In contrast, UCyA copolymers exhibited significantly less ordered morphologies with only first-order q maxima observed for the U_50_-*b*-(B_480_-*co-*U_20_)-*b*-U_50_ and U_50_-*b*-(B_450_-*co-*U_50_)-*b*-U_50_ and a loss of ordering for the U_50_-*b*-(B_420_-*co-*U_80_)-*b*-U_50_. Higher UCyA content in the central block of the copolymer increased the interaction of the UCy groups between in the central and external blocks, which disrupted the ordering for U_50_-*b*-(B_420_-*co-*U_80_)-*b*-U_50_ [36]. Both CyA and UCyA copolymers exhibited a decrease of domain spacing with increasing hydrogen bonds in the central block (Table 1), which also indicated a more mixed microstructure at higher hydrogen bonding compositions.

**Figure 6:**
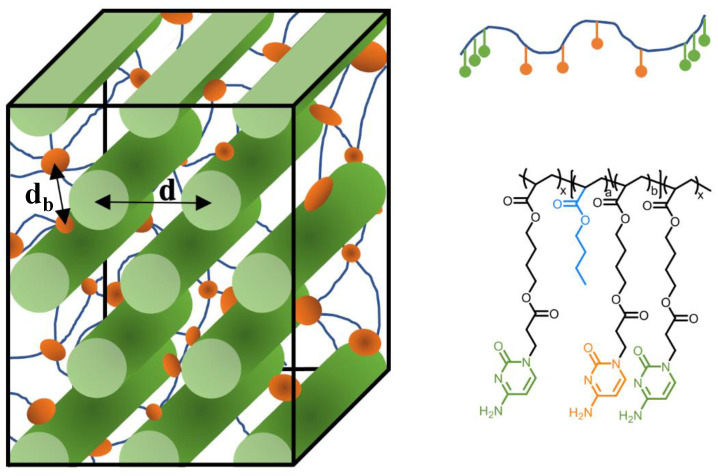
Schematic representation of the CyA triblock copolymer morphology based on SAXS.

At larger scattering vectors *q* > 1 nm^−1^, a broad scattering peak centered at *q*_b_ ~ 1.5 nm^−1^ remains relatively consistent for all CyA triblock copolymers. This peak was associated with the phase separated Cy domains in the central random block, as evidenced by the SAXS analysis of the B_450_-*co-*C_150_ random copolymer (Figure 5A). Additionally, the scattering peak became sharper as CyA in the central block increased, which was attributed to enhanced phase separation with increasing Cy incorporation. Figure 6 proposed a schematic representation of the CyA copolymer morphology according to the SAXS result. The large green dots represent the hard domains that are mainly composed of the external blocks. The characteristic dimension *d* described the distance between the hard domains and decreased with increasing CyA content (Table 1). The small orange dots represent CyA domains in the soft phase distributed in the *n*BA matrix with a characteristic dimension of *d*_b_ = 4.2 nm that was invariant to CyA concentration (Table 1). Atomic Force Microscope (AFM) (Figure S11) revealed a biphasic microstructure of C_50_-*b*-(B_450_-*co-*C_50_)-*b*-C_50_ where the light and the dark regions were attributed to the hard and soft phase, respectively. In contrast, the characteristic scattering peak of UCyA copolymers is less prominent at *q* > 1 nm^−1^ compared to the CyA copolymer, which confirmed the more mixed-phase morphology of UCyA copolymer. The stronger QHB association in the UCyA copolymer promoted the interactions between central and external blocks compared to the dimeric hydrogen bonding interactions in the CyA triblock copolymers. In fact, increased phase mixing in the UCyA triblock copolymer influenced the storage modulus above the second tan δ transition where the hard domains underwent QHB dissociation. The hard domain contained not only the external blocks but also the central blocks that were incorporated due to phase mixing. Increasing UCyA in the central block from C_50_-*b*-(B_480_-*co*-C_20_)-*b*-C_50_ to C_50_-*b*-(B_420_-*co*-C_80_)-*b*-C_50_ increased the physical crosslinks in the hard domain and thus enhanced the storage modulus from 1.9 to 22 MPa (Figure 4B). 

## 3. Materials and Methods

### 3.1. Materials

*n*-butyl acrylate (*n*BA, 99+%) was purchased from Sigma-Aldrich and passed through basic alumina columns before use. *α,α’*-Azobis-(isobutyronitrile) (AIBN, Fluka, 99%) was recrystallized twice from methanol. 1,4-butanediol diacrylate (Aldrich, 90%), cytosine (TCI, >98%), hexyl isocyanate (Aldrich, 97%), 2-(dodecylthiocarbonothioylthio)-2- methylpropionic acid (DDMAT, Sigma-Aldrich, 98%), *N*,*N’*-dicyclohexylcarbodiimide (Sigma-Aldrich, 99%), 4-(dimethylamino) pyridine (Sigma-Aldrich, ≥99%), potassium carbonate (anhydrous, Aldrich, 99%), and sodium sulfate (anhydrous, Spectrum, 99%) was used without further purification. Dimethyl sulfoxide (DMSO, HPLC grade), *N,N*-dimethylformamide (DMF, HPLC grade), dichloromethane (ACS grade), hexane (HPLC grade), and methanol (ACS grade) were purchased from Spectrum and used as received.

### 3.2. Synthesis of Poly(nBA-co-CyA) Difunctional Macro-CTA (Scheme 1)

Poly(*n*BA-*co*-CyA) was prepared using RAFT polymerization. Difunctional CTA, 1,6-bis(DDMAT)-hexane diamide (diDDMAT-NH_2_), and CyA was synthesized according to our previous literature [34]. In a typical random copolymer synthesis, a 100-mL, single-necked, Schlenk flask was charged with diDDMAT-NH_2_ (25.2 mg, 0.031 mmol, 1 eq), *n*BA (2 g, 11.89 mmol, 501 eq), CyA (0.22 g, 0.71 mmol, 23 eq), AIBN (0.5 mg, 0.0031 mmol, CTA/I = 10), and DMF (6.74 g, 25 *wt.* % solution). The solution was stirred at 70 °C for 24 h after subjecting to four freeze–pump–thaw cycles and refilling with argon. The copolymer was isolated from dialyzing in a CH_3_OH/CH_2_Cl_2_ mixture for two days and subsequent drying under vacuum at 60 °C overnight to afford a light yellow tacky solid. ^1^H NMR spectroscopy in CD_3_Cl/DMSO-*d_6_* revealed the degree of polymerization (DP) of each unit: 20 for CyA and 488 for *n*BA (Table S1, Figure S1).

### 3.3. Reactivity Ratio Determination

Fineman-Ross and Kelen-Tüdös presented methods to calculate the reactivity ratio values (*r_nBA_* and *r_CyA_*), and thus, a variety of different monomer feed ratios (*f_CyA_*) were explored. Limiting the copolymerization at a low conversion (<10%) allowed to determine the instantaneous copolymer composition (*F_CyA_*) [32]. Copolymerization equation (Equation (1)) correlated the monomer feed (*f_CyA_* and *f_nBA_*) and copolymer composition (*F_CyA_* and *F_nBA_*) to the reactivity ratios (*r_CyA_* and *r*_nBA_**)

(1) $$F_{CyA}=\frac{r_{CyA}f_{CyA}^{2}+f_{CyA}f_{nBA}}{r_{CyA}f_{CyA}^{2}+2f_{CyA}f_{nBA}+r_{nBA}f_{nBA}^{2}}$$

Fineman-Ross method rearranged Equation (1) to afford linearization expression of reactivity ratios (Equation (2)).

(2) $$\frac{f_{1}\left(2F_{1}-1\right)}{\left(1-f_{1}\right)F_{1}}=\frac{f_{1}^{2}\left(1-F_{1}\right)}{{\left(1-f_{1}\right)}^{2}F_{1}}r_{1}-r_{2}$$

where *G* and *H* are

$$G=\frac{f_{1}\left(2F_{1}-1\right)}{\left(1-f_{1}\right)F_{1}}$$

$$H=\frac{f_{1}^{2}\left(1-F_{1}\right)}{{\left(1-f_{1}\right)}^{2}F_{1}}$$

Therefore, Equation (2) can also be converted to

*G* = *Hr*_1_ − *r*_2_ (3)

where *G* and *H* were readily determined by monomer feed and copolymer composition at different monomer conversions calculated from ^1^H NMR spectroscopy. Based on Equation (3), a linear relationship was generated through plotting *G* against *H*, where −*r*_2_ and *r*_1_ corresponding to the intercept and slope of the linear relationship, respectively. However, Fineman-Ross method resulted in a local concentration of data points. To solve this problem, Kelen-Tüdös method distributed data points evenly through adding an arbitrary correction factor *α* to modify Equation (3) to Equation (4).

(4) η=⌊r1+r2α⌋ξ−r2α

where *η*, *ξ*, and *α* are

$$\eta =\frac{G}{\alpha +H}$$

$$\xi =\frac{H}{\alpha +H}$$

$$\alpha =\sqrt{H_{min}H_{max}}$$

Therefore, a new linear relationship was applied with −*r_nBA_*/α and *r_CyA_* corresponding to the intercept and slope of the curve, respectively.

### 3.4. Chain Extension of Poly(nBA-co-CyA) with CyA (Scheme 1)

A typical synthesis of poly(CyA-*b*-(*n*BA-*co*-CyA)-*b*-CyA) was conducted as follows: CyA (1.12 g, 3.64 mmol, 150 eq), AIBN (0.80 mg, 0.0048 mmol, CTA/I = 5), poly(*n*BA-*co*-CyA) macro-CTA (1.63 g, 0.024 mmol, 1 eq), and DMF (15.62 g, 15 *wt.* % solution) were charged into a single-necked Schlenk flask. The solution was stirred at 70 °C for 24 h after subjecting to four freeze-pump-thaw cycles and refilling with argon. ^1^H NMR spectroscopy in DMSO-*d_6_* determined a monomer conversion of 67%. The copolymer was isolated from dialyzing in a CH_3_OH/CH_2_Cl_2_ mixture for three days, subsequent drying under vacuum at 60 °C for overnight afforded a light-yellow solid. ^1^H NMR spectroscopy of the purified polymer in CD_3_OD/DMSO-*d_6_* revealed an average of 50 CyA units in each external block assuming an equal propagation rate on both chain ends (Figure S2, Table 1).

### 3.5. Synthesis of Poly(UCyA-b-(nBA-co-UCyA)-b-UCyA) Triblock Copolymers (Scheme 1)

Poly(UCyA-*b*-(*n*BA-*co*-UCyA)-*b*-UCyA) was prepared using post-functionalization of poly(CyA-*b*-(*n*BA-*co*-CyA)-*b*-CyA). In a representative triblock copolymer synthesis, 1 g (1 eq) of CyA block copolymer was dissolved in an anhydrous DMF/DMSO mixture (10 mL) in a sealed round-bottomed flask, followed by adding hexyl isocyanate (0.26 mL, 1.5 eq) dropwise using a syringe and then stirred at 20 °C for 20 min. The reaction flask was then reacted at 80 °C until a quantitative conversion of CyA to UCyA (indicated by ^1^H NMR spectroscopy) (Figure S3). The reaction was quenched by adding methanol into the solution, and the copolymer was isolated through dialysis in a CH_3_OH/CH_2_Cl_2_ mixture for two days and was then dried under reduced pressure at 60 °C for 18 h to afford a slightly yellow solid.

### 3.6. Polymer Film Preparation and Annealing Conditions

Triblock copolymer films for DMA, SAXS, and AFM were prepared by dissolving polymer samples in a mixture of DMSO and DMF at 6 *wt.* % concentration, and casting into poly(tetrafluoroethylene) molds to minimize deformation during solvent annealing and film removal. The molds were covered with a glass Petri dish and maintained at 50 °C for three days on a hot plate to allow slow evaporation of the solvents. The copolymer films were further dried in the vacuum at room temperature overnight and were then annealed at 100 °C for 12 h. The annealed films were stored in a desiccator prior to characterization.

### 3.7. Instrumentation

Proton nuclear magnetic resonance (^1^H NMR) spectroscopy was collected on an Agilent U4-DD2 400 Hz spectrometer at 23 °C in a mixture of CDCl_3_ and DMSO-*d_6_* (1:1, *v/v*). Thermogravimetric analysis (TGA) of cytosine and UCy-containing triblock copolymers was conducted on a TA Instruments TGA Q500 ramping from 30 °C to 550 °C under N_2_ purge at a heating rate of 10 °C/min. Differential scanning calorimetry (DSC) was performed on a TA Instruments DSC Q2000 using a heat/cool/heat cycle between −60 °C and 150 °C under N_2_ purge at a heating/cooling rate of 10 °C/min. Dynamic mechanical analysis (DMA) was conducted on a TA Instruments Q800 Dynamic Mechanical Analyzer in tension mode at a frequency of 1 Hz, an oscillatory amplitude of 5 µm, and a static force of 0.01 N. The annealed film was subjected to a temperature ramp of 3 °C/min, starting from −80 to 180 °C. An Instron 5500R universal testing instrument measured the uniaxial tensile properties of the compression-molded sample at a rate of 5 mm/min. The compression-molded film was cut using ASTMD-638-V cutter to generate tensile specimens. Tensile analysis data represented an average of five specimens with calculated standard deviations. Toughness was determined by calculating the area under the stress-strain curve.

Small angle X-ray scattering (SAXS) experiments were performed using a Rigaku S-Max 3000 3 pinhole SAXS system, equipped with a rotating anode emitting X-ray with a wavelength of 0.154 nm (Cu Kα). The sample-to-detector distance was 1600 mm, and the q-range was calibrated using a silver behenate standard. Two-dimensional SAXS patterns were obtained using a 2D multiwire, proportional counting, gas-filled detector, with an exposure time of 2 h. The SAXS data were corrected for sample thickness, transmission, and background, and were put on an absolute scale by correction using a glassy carbon standard from the Advanced Photon Source (APS). All the SAXS data were analyzed using the SAXSGUI software package to obtain radially integrated SAXS intensity versus the scattering vector q, where q = (4π/λ) sin (θ), θ is one half of the scattering angle and λ is the X-ray wavelength. The scattering profiles were vertically shifted to facilitate a comparison of peak positions. Domain spacing was calculated according to the equation d = 2π/q. Tapping-mode AFM imaging was performed using a Veeco MultiMode scanning probe microscope, at a set-point ratio of 0.3 with a magnification of 1 × 1 μm. Phase images were mapped using Veeco’s Nanosensor silicon tips with a spring constant of 42 N m^−1^.

## 4. Conclusions

Synthesis of CyA and UCyA-containing A-AB-A triblock copolymers provided a strategy to design high-performance TPEs. Manipulation of hydrogen bonding strength and distribution enabled tunable mechanical properties and bulk morphology. Triblock copolymers containing weakly associated Cy units formed hexagonally packed cylindrical morphologies, whereas the UCy units with highly oriented and stronger QHB association promoted phase mixing, resulting in less-ordered microstructures in the UCyA copolymers. Surprisingly, such phase mixing did not impair the mechanical properties of UCyA copolymers. On the contrary, the UCyA copolymer with strong QHB interactions retained the three-dimensional structure of the copolymer film until 150 °C. Additionally, the topological design endowed A-AB-A UCyA copolymer with exceptional tensile properties compared to their random and A-B-A triblock copolymer counterparts. Balancing these design parameters in concert provides a roadmap for tuning the thermomechanical properties and morphologies of supramolecular TPEs.

## Figures and Tables

**Table 1:** Compositions, thermal properties, and morphological properties of CyA and UCyA copolymers with varying amounts of hydrogen bonding contents.

Sample Name	CyA/UCyA in Copolymer (mol %)	DP ^a^	T_d, 15 *wt.*__%_^b^ (°C)	T_g1_^c^ (°C)	T_g2_^c^ (°C)	d ^d^ (nm)	d_b_^d^ (nm)
C_50_-*b*-(B_480_-*co-*C_20_)-*b*-C_50_	20	50–488/20–50	326	−37	85	41	N/A
C_50_-*b*-(B_450_-*co-*C_50_)-*b*-C_50_	25	51–453/50–51	317	−23	86	38	4.2
C_50_-*b*-(B_420_-*co-*C_80_)-*b*-C_50_	30	51–418/78–51	312	6	83	32	4.2
U_50_-*b*-(B_480_-*co-*U_20_)-*b*-U_50_	20	50–488/20–50	280	−35	N/A	41	N/A
U_50_-*b*-(B_450_-*co-*U_50_)-*b*-U_50_	25	51–453/50–51	265	−24	N/A	31	4.5
U_50_-*b*-(B_420_-*co-*U_80_)-*b*-U_50_	30	51–418/78–51	262	−9	N/A	N/A	4.4

^a 1^H NMR spectroscopy. ^b^ TGA, 10 °C min^−1^,23–600 °C, N_2_. ^c^ DSC, 10 °C min^−1^, −60–50 °C, N_2_. ^d^ SAXS, λ = 0.154 nm (Cu, K_α_), and 23 °C.

**Table 2:** Summary of tensile testing derived from the stress-strain curve (Figure S8).

Sample	Young’s Modulus (MPa)	Strain at Break (%)	Stress at Break (MPa)	Toughness (MJ/m^−3^)
B_450_-*co*-U_150_	31 ± 2	89 ± 10	7.0 ± 0.5	4.3 ± 0.6
U_50_-*b*-(B_450_-*co-*U_50_)-*b*-U_50_	23 ± 4	131 ± 10	9.1 ± 0.3	8.6 ± 0.7
U_75_-*b*-B_450_*-b*-U_75_	N/A			

## Data Availability

The data presented in this study are available in insert article or Supplementary Materials.

## Supplementary Materials

The following are available online, Figure S1: ^1^H NMR spectroscopy of poly(CyA-*co*-*n*BA) macro-CTA. (A) The crude product in DMSO-*d*_6_ from reaction solution for conversion calculation and (B) purified polymer in DMSO-*d*_6_ + CDCl_3_ for determining molecular weight, Figure S2: ^1^H NMR spectroscopy of purified C_50_-*b*-(B_480_-*co-*C_20_)-*b*-C_50_ in DMSO-*d*_6_ + CDCl_3_, Figure S3: ^1^H NMR spectroscopy of the purified C_50_-*b*-(B_480_-*co-*C_20_)-*b*-C_50_ and U_50_-*b*-(B_480_-*co-*U_20_)-*b*-U_50_ in DMSO-*d*_6_ + CDCl_3_, calculation of monomer conversion, Figure S4: Determination of reactivity ratios for the copolymerization of CyA and *n*BA using the Fineman−Ross method, Figure S5: Fraction of CyA segment in the polymer as a function of overall conversion. Figure S6: Dynamic mechanical temperature ramps of storage modulus and tan δ of the solution cast C_50_-*b*-(B_450_-*co-*C_50_)-*b*-C_50_ and B_450_-*co-*C_50_, Figure S7: Dynamic mechanical temperature ramps of storage modulus and tan δ of the solution cast U_50_-*b*-(B_450_-*co-*U_50_)-*b*-U_50_ and B_450_-*co-*C_150_, Table S1: Compositions and thermal properties of CyA and UCyA copolymer macro-CTA with varying amounts of hydrogen bonding content, Figure S8: Stress-strain curves of B_450_-*co*-U_150_ and U_50_-*b*-(B_450_-*co*-U_50_)-*b*-U_50_, Figure S9: The cracked film of U_75_-*b*-B_450_-*b*-U_75_ during thermal annealing, Figure S10: Dynamic mechanical temperature ramps of storage modulus and tan δ of the solution cast C_50_-*b*-(B_420_-*co-*C_80_)-*b*-C_50_ and B_420_-*co-*C_80_, Figure S11: AFM phase image for the solution casted C50-b-(B450-co-C50)-b-C50 film.

## Sample Availability

Samples of CyA monomer, CyA triblock copolymers, and UCyA triblock copolymers are available from the authors.
