# Supplementary material for: Quadruple Hydrogen Bond-Containing A-AB-A Triblock Copolymers: Probing the Influence of Hydrogen Bonding in the Central Block
Source: Molecules. 2021 Aug 3;26(15):4705. doi: 10.3390/molecules26154705 (PMC8348091; doi:10.3390/molecules26154705)
Supplement: Supplementary file 1 [file molecules-26-04705-s001.zip › molecules-1317812-supplementary.pdf]

Quadruple Hydrogen Bond-Containing A-AB-A triblock Copolymers: Probing the Influence of Hydrogen Bonding in the Central Block

Boer Liu[1], Xi Chen[2], Glenn A. Spiering[2], Robert B. Moore[2], and Timothy E. Long[1]*

[1]*Biodesign Center for Sustainable Macromolecular Materials and Manufacturing, School of Molecular Sciences*

Arizona State University, Tempe AZ 85281, USA

[2]*Department of Chemistry, Macromolecules Innovation Institute (MII), Virginia Tech, Blacksburg, VA 24061, USA*

*To whom correspondence should be addressed: E-mail: Timothy.E.Long@asu.edu.

Supplementary Materials

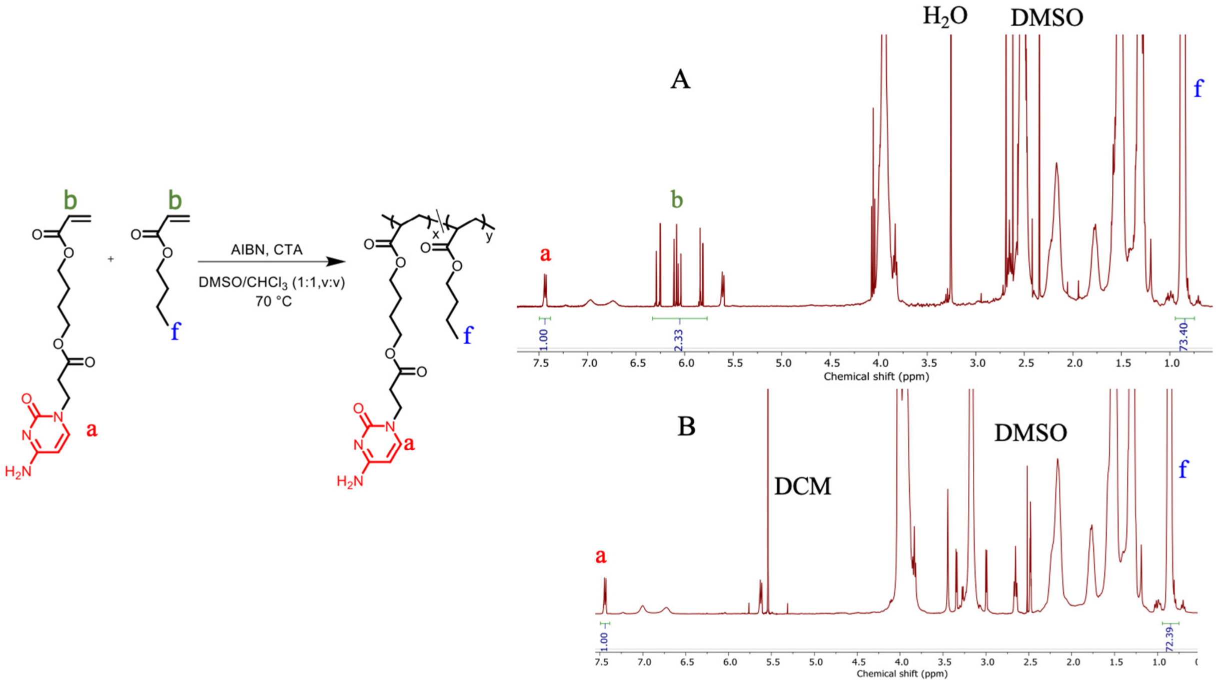

Figure S1. ^{1}H NMR spectroscopy of poly(CyA-*co*-*n*BA) macro-CTA. (A) The crude product in DMSO-d_6 from reaction solution for conversion calculation and (B) purified polymer in DMSO-d_6 + $CDCl_3$ for determining molecular weight.

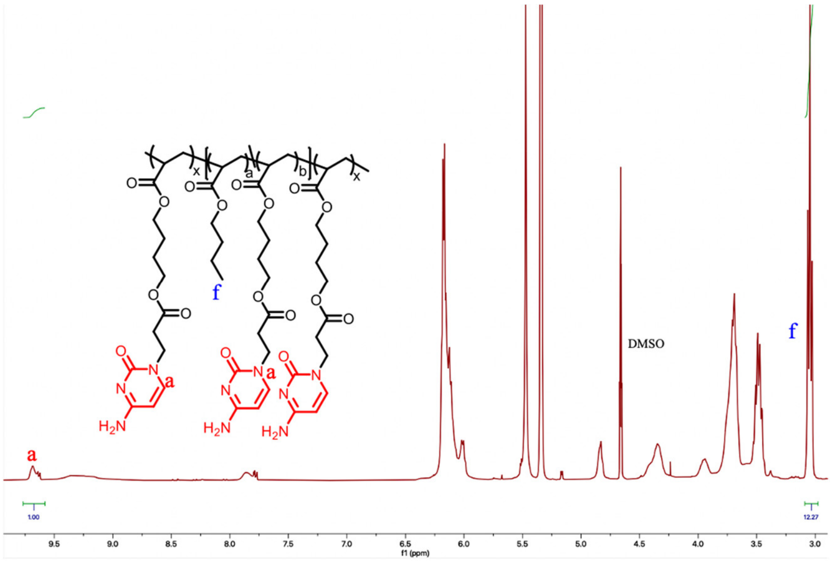

Figure S2. ^{1}H NMR spectroscopy of purified C_{50}-*b*-(B_{480}-*co*-C_{20})-*b*-C_{50} in DMSO-d_6 + $CDCl_3$.

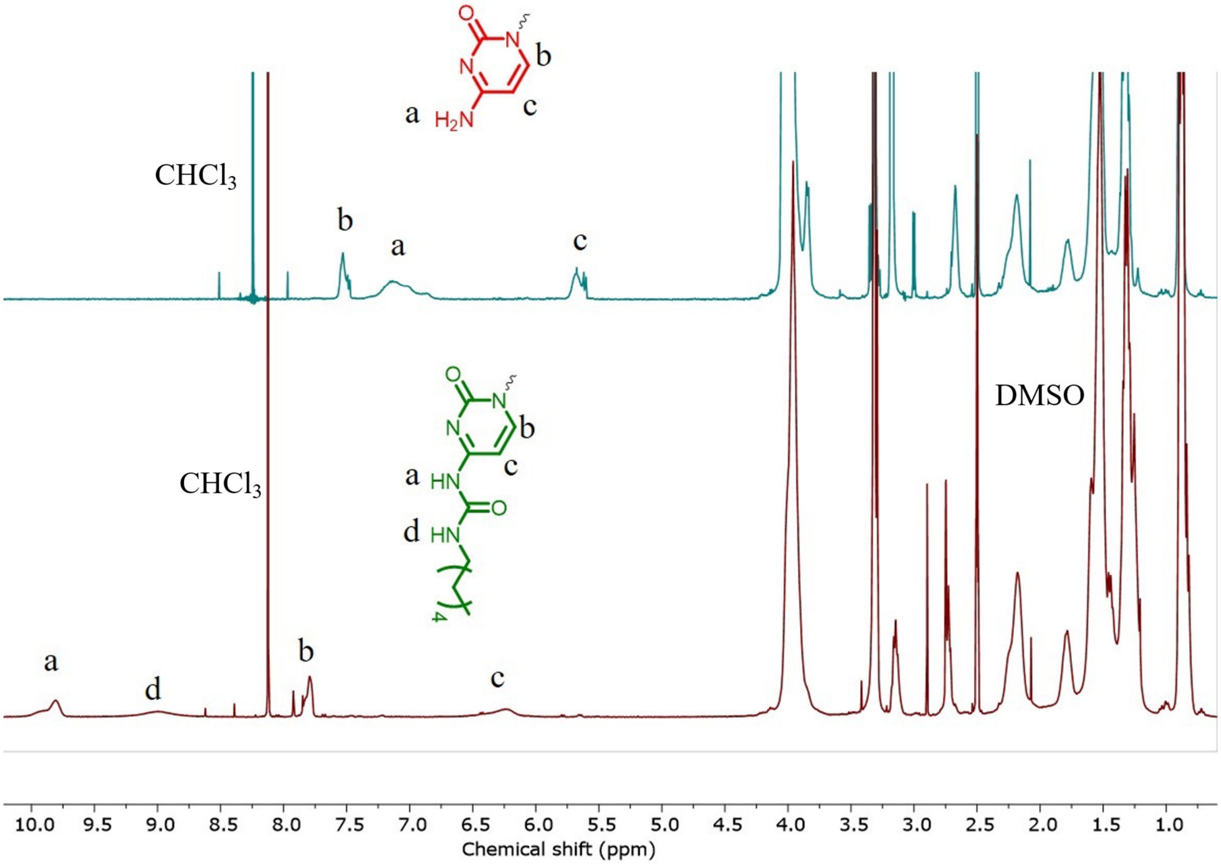

Figure S3. ^{1}H NMR spectroscopy of the purified C_{50}-*b*-(B_{480}-*co*-C_{20})-*b*-C_{50} and U_{50}-*b*-(B_{480}-*co*-U_{20})-*b*-U_{50} in DMSO-d_6 + $CDCl_3$.

Calculation of Monomer Conversion

$$A = \frac{DP_{CyA}}{DP_{CyA}+DP_{nBA}} \tag{S1}$$

$$B = \frac{Feed_{CyA}}{Feed_{CyA}+Feed_{nBA}} \tag{S2}$$

$$C = \frac{DP_{CyA}+DP_{nBA}}{Feed_{CyA}+Feed_{nBA}} \tag{S3}$$

where A and B are the molar percentage of CyA in the copolymer and feed, respectively (**Table S1**). C stands for the overall conversion of the monomer (**Table S1**).

Take the ratio of eq. S1 and S2 yielded eq S4:

$$\frac{A}{B} = \frac{DP_{CyA}}{Feed_{CyA}} \frac{Feed_{CyA}+Feed_{nBA}}{DP_{CyA}+DP_{nBA}} = conv_{CyA}\left({}^{1}\!/_{C}\right) \tag{S4}$$

where the ratio of DP_{CyA} and $Feed_{CyA}$ is the conversion of CyA, $conv_{CyA}$.

Therefore, rearranging eq. S4 affords the expression of $conv_{CyA}$ (eq S5):

$$conv_{CyA} = \frac{A \times C}{B} \tag{S5}$$

Similarly, the expression of *n*BA conversion is shown in eq S6:

$$conv_{nBA} = \frac{(1-A) \times C}{1-B} \tag{S6}$$

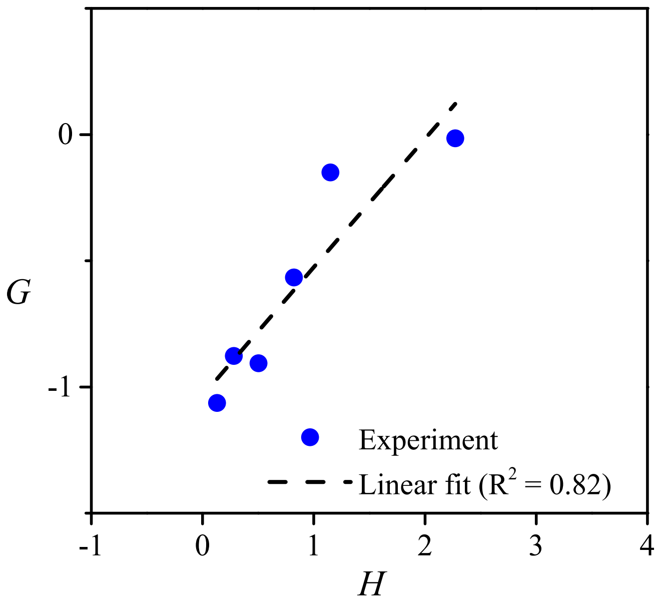

Figure S4. Determination of reactivity ratios for the copolymerization of CyA and *n*BA using the Fineman−Ross method.

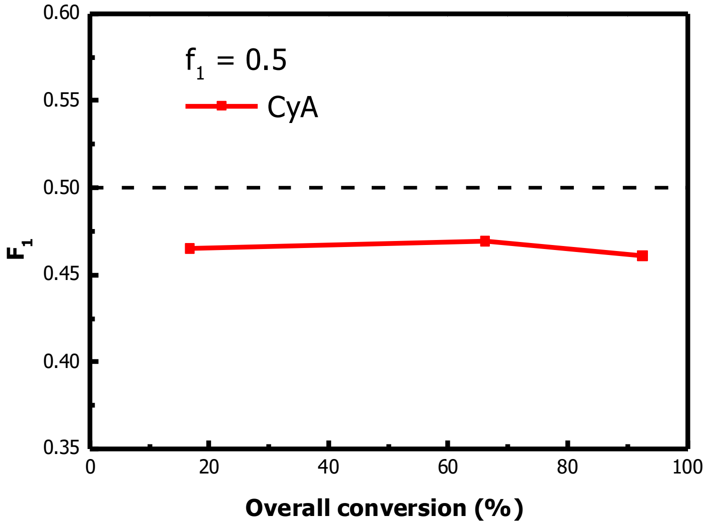

Figure S5. Fraction of CyA segment in the polymer as a function of overall conversion.

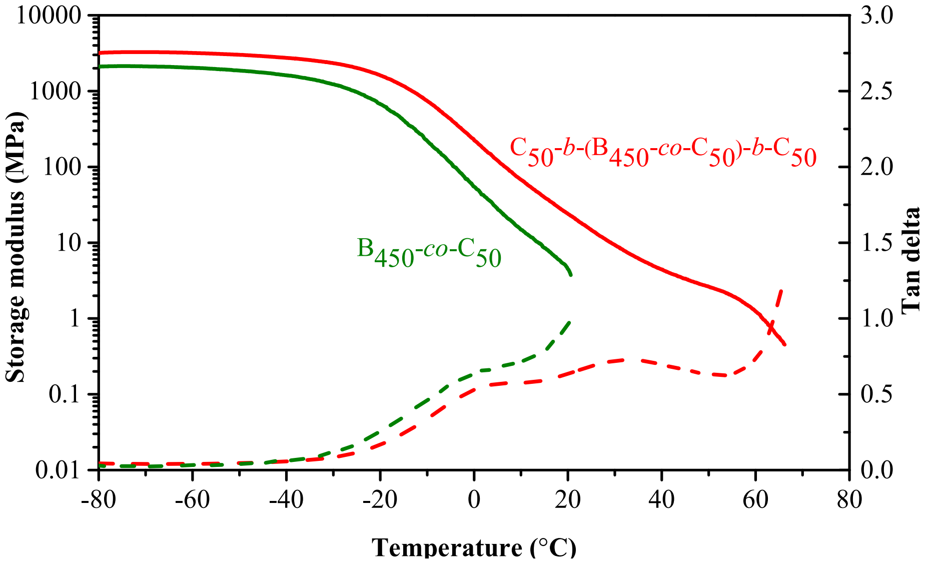

Figure S6. Dynamic mechanical temperature ramps of storage modulus and tan δ of the solution cast C_{50}-*b*-(B_{450}-*co*-C_{50})-*b*-C_{50} and B_{450}-*co*-C_{50}.

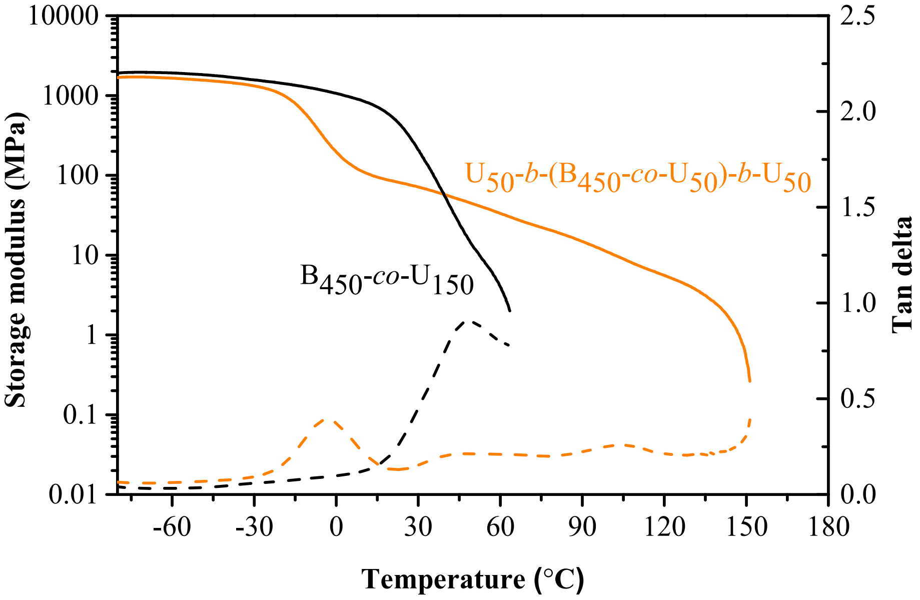

Figure S7. Dynamic mechanical temperature ramps of storage modulus and tan δ of the solution cast U_{50}-*b*-(B_{450}-*co*-U_{50})-*b*-U_{50} and B_{450}-*co*-C_{150}.

Table S1. Compositions and thermal properties of CyA and UCyA copolymer macro-CTA with varying amounts of hydrogen bonding content

	CyA in feed, f_{CyA} (mol %)	CyA/UCyA in copolymer, F_{CyA} (mol %)[a]	Overall conversion [a] (%)	Feed	DP[a]	T_{g1}[b] (°C)
B_{480}-*co*-C_{20}	4	4	97	501/23	488/20	-38
B_{450}-*co*-C_{50}	14	10	86	505/79	453/50	-23
B_{420}-*co*-C_{80}	22	16	82	473/131	418/78	0
B_{480}-*co*-U_{20}	N/A	4	N/A	N/A	488/20	-39
B_{450}-*co*-U_{50}	N/A	10	N/A	N/A	453/50	-26
B_{420}-*co*-U_{80}	N/A	16	N/A	N/A	420/78	-10

[a] ^{1}H NMR spectroscopy

[b] DSC, 10 °C min^{-1}, −60 – 150 °C, N_2.

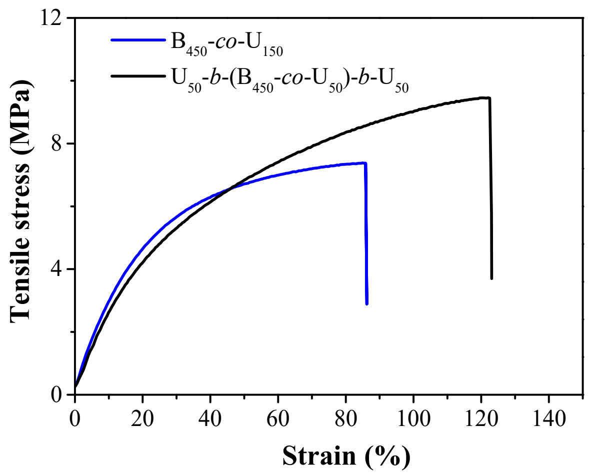

Figure S8. Stress-strain curves of B_{450}-*co*-U_{150} and U_{50}-*b*-(B_{450}-*co*-U_{50})-*b*-U_{50}.

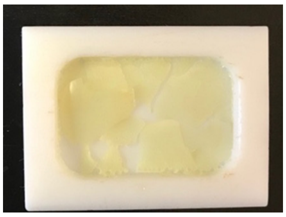

Figure S9. The cracked film of U_{75}-*b*-B_{450}-*b*-U_{75} during thermal annealing.

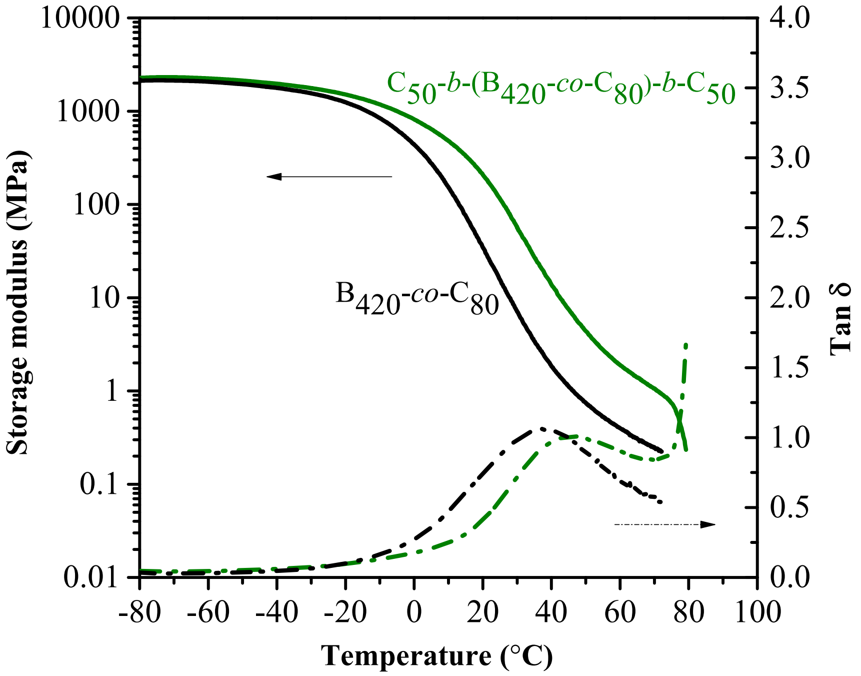

Figure S10. Dynamic mechanical temperature ramps of storage modulus and tan δ of the solution cast C_{50}-*b*-(B_{420}-*co*-C_{80})-*b*-C_{50} and B_{420}-*co*-C_{80}

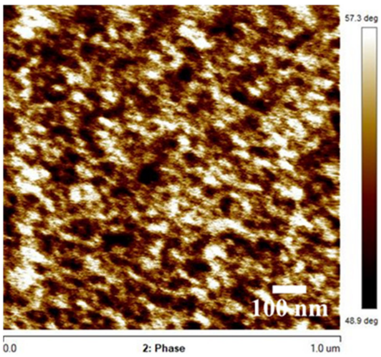

Figure S11. AFM phase image for the solution casted C_{50}-*b*-(B_{450}-*co*-C_{50})-*b*-C_{50} film.
